# MUSTN1 Interaction With SMPX Regulates Muscle Development and Regeneration

**DOI:** 10.1111/cpr.13809

**Published:** 2025-01-19

**Authors:** Yu Fu, Xin Hao, Peng Shang, Jingru Nie, Yangzom Chamba, Bo Zhang, Hao Zhang

**Affiliations:** ^1^ Frontiers Science Center for Molecular Design Breeding (MOE) China Agricultural University Beijing China; ^2^ National Engineering Laboratory for Livestock and Poultry Breeding, Beijing Key Laboratory of Animal Genetic Engineering China Agricultural University Beijing China; ^3^ Department of Animal Husbandry Xizang Agricultural and Animal Husbandry University Linzhi China

**Keywords:** muscle development, MUSTN1, regeneration, SMPX

## Abstract

Pigs are important agricultural animals whose growth rate and meat production performance are related to muscle development. Musculoskeletal embryonic nuclear protein 1 (MUSTN1) participates in various biological processes, including myogenesis and growth in animals, but the physiological functions and mechanisms of porcine MUSTN1 on muscle development are unclear; thus, we aimed to elucidate them. We found that MUSTN1 was highly expressed in the muscles of fast‐growing pigs. Functionally, *MUSTN1* promoted myoblast proliferation and differentiation. *MUSTN1* knockout mice exhibited reduced muscle mass and fibre cross‐sectional area, decreased exercise endurance, and delayed muscle regeneration. Small muscle protein X‐linked (SMPX) was identified as an interacting protein of MUSTN1, and its promotion of myogenic differentiation depended on MUSTN1. Furthermore, MUSTN1 stabilised SMPX and maintained myofiber morphology. This study suggests that *MUSTN1* is a critical regulator in the control of muscle development and regeneration and is a potential target for animal genetic improvement and the treatment of human muscle disease.

## Graphical abstract

**Model of the role of MUSTN1 in skeletal muscle development.** *MUSTN1*‐KO mice display delayed muscle growth and regeneration. MUSTN1 protects SMPX from instability and maintains the morphology of muscle fibres. MUSTN1 and SMPX synergistically promote myoblast proliferation and dependently facilitate differentiation.

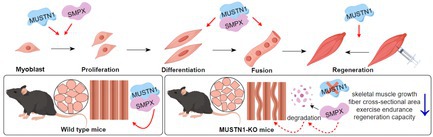

## 1 Introduction

Skeletal muscle, the most abundant tissue in humans and animals, plays a key role in health and daily life. Impaired muscle development and regeneration can lead to muscle diseases such as sarcopenia, dysplasia, and muscle metabolism disorders [1, 2, 3]. Skeletal muscle growth rate and weight are critical economic traits in agricultural animal production that determine meat yield and quality [4]. Skeletal muscle development, also known as myogenesis, is a complex process involving embryonic development, muscle regeneration, and muscle repair [5]. Muscle precursor cells undergo essential processes, including proliferation, migration, differentiation, and fusion, to form multinucleated myotubes, which are strictly regulated by various genes [6].

Musculoskeletal embryonic nuclear protein 1 (*MUSTN1*), known as Mustang, has been found in a rat femur fracture model and is significantly expressed in muscle and skeletal tissues and involved in various biological processes such as muscle and cartilage development [7, 8]. Differentially expressed *MUSTN1* has been identified in muscle RNA‐seq data from sheep [9], yak [10], chickens [11, 12], donkeys [13], and mink [14]. *MUSTN1* is highly expressed in activated satellite cells, primary myoblasts, and neonatal muscle ducts [15]. A higher expression level of *MUSTN1* was observed in skeletal muscle tissues of broilers than in those of layers during post‐hatch at 2–8 weeks [16]. During embryonic development, quail exhibited faster muscle development and higher expression of *MUSTN1* gene in the breast muscle compared to chicken [17]. The promoter region of *MUSTN1* in ducks was found to contain multiple transcription factor‐binding sites involved in the regulation of muscle development [18]. These findings indicate that *MUSTN1* may play a role in regulating skeletal muscle growth and hypertrophy in animals. Indeed, *MUSTN1* interference inhibited differentiation and fusion of C2C12 cells [19] and promoted apoptosis of skeletal muscle satellite cells in chicken [20]. In humans, findings showed that exercise may increase the expression level of *MUSTN1* and result in hypertrophic muscle [21]. Clinically, *MUSTN1* expression was potentially linked to muscle‐wasting conditions such as muscular dystrophies [22]. Furthermore, Han et al. [23] found a mutation site of *MUSTN1* in pigs (265C>T) that was significantly associated with average daily gain. Pigs are an important agricultural economic animal and human disease research model. However, most of the reports on *MUSTN1* in muscles remain at the level of high‐throughput sequencing and gene expression, with few using preliminary cell validation and many focusing on poultry. Thus, elucidating the regulatory role and underlying molecular mechanisms of porcine *MUSTN1* in muscle development is necessary.

In this study, we investigated the expression of *MUSTN1* in the muscle tissues of pigs with different growth rates. The functions of *MUSTN1* in the regulation of myoblast proliferation, differentiation, muscle growth, and regeneration were identified using in vitro tests and *MUSTN1* knockout (*MUSTN1*‐KO) mice. We identified a small muscle protein X‐linked (SMPX), that interacts with MUSTN1, participating in regulating myogenesis. Our findings indicate that *MUSTN1* is critical for skeletal muscle growth and regeneration and provides a theoretical basis for improving the growth performance of animals and treating human muscle diseases.

## 2 Methods

### 2.1 Experimental Materials

All tissues were sampled from three pig breeds with different growth rates: Yorkshire (YY), a commercial breed with fast growth and large size, and Tibetan (TP) and Wujin (WJ), small‐ and medium‐sized slow‐growing indigenous Chinese breeds, respectively. All pigs were grown at the Xizang Agricultural and Animal Husbandry University. Nine embryos from each group were collected from two pregnant shows 60 days after insemination for RNA extraction. *Longissimus dorsi* (LD) muscle tissues from the 12th rib were sampled.

### 2.2 Total RNA Preparation and Gene Expression Analysis

Total RNA was extracted using TRIzol reagent (Invitrogen, Carlsbad, CA, USA) according to a routine protocol and reverse‐transcribed to cDNA by using the FastQuant Reverse Transcriptase Kit (TIANGEN, Beijing, China). Gene expression was assessed using semi‐quantitative polymerase chain reaction (PCR) and quantitative real‐time PCR (qRT‐PCR). Semi‐quantitative PCR was performed as described in an existing study [24], and qRT‐PCR was performed using SYBR Green Master Mix (TIANGEN) according to the manufacturer's instructions. Analyses were performed using the relative quantification Delta–delta Ct method [25] with gene‐specific primers (Table S1). *β*‐*actin* and *GAPDH* were used as housekeeping genes to normalise all data.

### 2.3 Cell Culture

C2C12 cells were grown in incubators at 37°C and 5% CO_2_ and cultured in Dulbecco's Modified Eagle's Medium (DMEM; Gibco, Grand Island, NY, USA) containing 10% fetal bovine serum (FBS, Gibco) and 1% penicillin–streptomycin (PS, Gibco). For the induction of myogenic differentiation, the cells were transferred to DMEM supplemented with 2% horse serum (HS, Gibco) when they reached 80%–90% confluence.

### 2.4 Isolation and Culture of Primary Myoblasts

The hind limb muscles of 6‐week‐old male mice and LD muscles of 7‐day‐old‐boar were minced and digested using 0.2% type II collagenase (Sigma–Aldrich, St. Louis, MO, USA) and 2.5 U/mL dispase (Roche Applied Science, Nutley, NJ, USA) for 1 h. The slurry was filtered through 40 μm cell strainers. The cell suspension was centrifuged (1500 × *g*, 5 min, 25°C) and cultured in Ham's F10 nutrient mixture medium (Gibco) supplemented with 20% FBS, 1% PS, and 5 ng/mL basic fibroblast growth factor (PeproTech, Cranbury, NJ, USA). Primary myoblasts were seeded on collagen‐coated cell culture plates.

### 2.5 Plasmid Construction, siRNA Synthesis, and Cell Transfection

Overexpression plasmids pCDH‐*MUSTN1*‐pig, pCDH‐*MUSTN1*‐mouse, pcDNA3.1‐*MUSTN1*‐mouse‐3×FLAG, and pcDNA3.1‐*SMPX*‐mouse‐3×FLAG were constructed using F/R primers (Table S2). *MUSTN1* and scrambled siRNAs were synthesised according to the manufacturer's instructions (GenePharma, Shanghai, China). The sequences of mouse‐*MUSTN1* siRNAs are shown in Table S3. For cell transfection, C2C12 cells and myoblasts were transfected with 4 μg of the expression plasmid or 10 μL siRNAs for 4–6 h using Lipofectamine 2000 in each well of a 6‐well plate, followed by incubation in growth or differentiation medium.

### 2.6 Cell Proliferation Assays

Cell proliferation was measured using 5‐ethynyl‐2‐deoxyuridine (EdU) (RiboBio, Guangzhou, China) and Cell counting kit‐8 (CCK8) (Beyotime Biotechnology). For EdU staining, cells were incubated with 50 mM EdU for 2 h before fixation and permeabilization. Finally, the cells and nuclei were stained using EdU and Hoechst solutions for 30 min. For CCK8, cells were incubated with 10 μL CCK8 for 1 h in 96‐well plates, and absorbance was measured to detect cell proliferation.

### 2.7 Cell Migration Assays

Cell migration was measured using cell wound healing and Transwell assays as described in an existing study [26]. After cell transfection, a linear scratch was created in the monolayer of cells by using a sterile 200 μL micropipette tip, and the detached cells were washed with PBS (Gibco) three times. The remaining cells were incubated in DMEM containing 2% FBS for 24 h, and the wound area and cell migration were imaged under a microscope (ZEISS, Jena, Germany). For the Transwell assay, cells were serum‐starved in serum‐free medium for 6–8 h and then placed in the top chamber. The lower chamber was filled with DMEM containing 10% FBS. After 12 h, non‐migrating cells in the upper chambers were removed using a cotton swab. Cells that migrated to the lower surfaces of the inserts were stained using 0.1% crystal violet and observed under a microscope (ZEISS).

### 2.8 Western Blot

Total protein lysates were extracted from myoblasts and skeletal muscles and resolved using SDS‐PAGE. The proteins were incubated with primary antibodies against anti‐MUSTN1 (ABD115, 1:500, Sigma–Aldrich), anti‐MyoG (ab1835, 1:500, Abcam, Cambridge, United Kingdom), anti‐MyHC (M4276, 1:500, Sigma–Aldrich), anti‐β‐actin (sc‐4777, 1:1000, Santa Cruz Biotechnology, California, USA), anti‐SMPX (A6744, 1:500, ABclonal, Wuhan, China), anti‐His (AE003, 1:5000, ABclonal), anti‐GST (AE001, 1:5000, ABclonal), anti‐FLAG (20543‐1‐AP, 1:1000, Proteintech, Chicago, USA), and anti‐Histone H3 (A2348, 1:5000, ABclonal). Protein levels were normalised to those of the housekeeping proteins β‐actin or histone H3, and densitometric quantification of the western blot bands was performed using ImageJ software (National Institutes of Health, Bethesda, MD, USA).

### 2.9 Immunofluorescence Staining

The cells and tissue sections were fixed with 4% paraformaldehyde for 15 min at 25°C, washed thrice with PBS, and blocked with goat serum for 1 h. The primary antibodies used were as follows: anti‐MyHC (M4276, 1:200, Sigma‐Aldrich), anti‐eMyHC (F1.652, 1:50; Developmental Studies Hybridoma Bank, Lowa, USA), anti‐laminin (L9393, 1:1000, Sigma–Aldrich), anti‐SMPX (A6744, 1:100, ABclonal), and anti‐FLAG (20543‐1‐AP, 1:100, Proteintech). Secondary antibodies (SA00013‐2 and SA00013‐3, 1:400, Proteintech) were added after washing thrice with PBS. Nuclei and F‐actin were stained using DAPI (Roche) and phalloidin (ab176757, Abcam), respectively, according to the manufacturer's instructions. Images were captured using a confocal microscope (Leica image analysis system, Model Q500MC, Weztlar, Germany).

### 2.10 Generation of MUSTN1 Knockout Mice and Phenotype Measurements

*MUSTN1*‐KO mice were purchased from Cyagen Biosciences (Guangzhou, China). The selected gRNAs (Table S4) were used to delete the 2207 bp genomic region of *MUSTN1*. All mice were in the C57BL/6N background and housed in a pathogen‐free facility: controlled temperature (22°C ± 1°C) and humidity (60% ± 10%), a 12 h light/dark cycle, water *ad libitum*, and a normal diet. Genotypes were determined using PCR from tail DNA and the primers listed in Table S4. Wild‐type (WT) mice had an amplicon of 606 bp, and KO mice had an amplicon of 409 bp. The founder mice were randomly mated to produce offspring for additional studies, and their body weight and length were measured monthly. Muscles and visceral organs of KO and WT mice aged 1–4 months were collected and weighed. All experimental animal procedures were performed in accordance with the China Agricultural University's regulations for animal care and handling. The China Agricultural University Laboratory Animal Welfare and Animal Experimental Ethics Committee (permit number: AW80203202‐1‐1) authorised the study and approved the protocol.

### 2.11 Muscle Injury and Evan's Blue Dye Uptake

Cardiotoxin (CTX) (Sigma–Aldrich) injury induction and muscle regeneration assays were performed as described in an existing study [27]. In brief, 50 μL CTX (20 μM) was injected into the tibialis anterior (TA) muscles of 2‐month‐old mice. Evans blue dye (10 mg/mL in PBS) was injected intraperitoneally (0.1 mL per 10 g body weight) into mice for 18 h before killing. TA muscles harvested at 3, 5, 7, and 14 days after injury were fixed in 4% paraformaldehyde. The sections were examined under a microscope.

### 2.12 Histology Staining

Skeletal muscle samples, with or without injury, were collected, fixed in 4% paraformaldehyde, and processed for routine paraffin histology. Muscle sections were prepared and stained using Haematoxylin and Eosin (H&E) and Masson's trichrome stain to assess tissue morphology. Sections were examined under an inverted microscope (ZEISS). Skeletal muscles were fixed in glutaraldehyde (Solarbio, Beijing, China), and the morphology of the muscle fibres was observed using transmission electron microscopy.

### 2.13 Exhaustive Swimming Experiment

Swimming sessions were performed in separate cylindrical tanks with a water temperature of 28°C. The amount of water ensured that the mouse tails did not touch the bottom. Unloaded mice swam for 10–30 min/day for weekly adaptive swimming training. Before each formal session, a load corresponding to 7% of body weight was applied to the mouse tails. After fasting overnight (15 h), the mice were simultaneously placed in the swimming tank side by side for the acute swimming test protocol. The time at which the mice became exhausted was recorded. Exhaustion was defined as remaining underwater for more than 5 s.

### 2.14 Magnetic Resonance Imaging

Magnetic resonance imaging (MRI) measurements were performed under isoflurane anaesthesia (concentration, 2%) by using an MRI system equipped with a 7 T/16 cm wide‐bore instrument. Twenty contiguous coronal slices (thickness = 1 mm) were acquired, covering the region from the knee to the ankle. The scan parameters were as follows: echo time (TE) = 7.8 ms; repetition time (TR) = 500 ms; field of view = 100 × 60 mm^2^; matrix size = 256 × 256; acquisition time = 20 min. Images were analysed using VNMRJ 4.0.

### 2.15 Co‐Immunoprecipitation Assay

Cells were harvested 48 h after transfection with pcDNA3.1‐*MUSTN1*/*SMPX* (mouse)‐3×FLAG and an empty vector. The cells were lysed on ice by using a lysis buffer (RM00022, ABclonal) with a protease inhibitor cocktail (RM02916, ABclonal). After centrifugation at 2000 rpm for 5 min at 4°C, the samples were mixed with 20 μL anti‐FLAG magnetic beads. The beads were continuously rotated with the lysate overnight at 4°C, washed three to five times with lysis buffer, and the solution was subjected to magnetic separation. Protein loading buffer was added, and the mixture was boiled for 10 min. Western blotting was performed to determine whether the final immunocomplex supernatant contained enriched prey proteins.

### 2.16 Protein Purification and In Vitro Pull‐Down

The recombinant plasmids pGEX‐4T‐1‐*MUSTN1*, pET‐30a‐*SMPX*, and empty pGEX‐4T‐1 were transformed into *Escherichia coli* strain BL21 (DE3) cells, and the primers are shown in Table S5. Bacteria were grown to an OD_600_ of 0.8 at 37°C and induced with 1 mM isopropyl‐β‐D‐thiogalactoside (IPTG) overnight at 16°C. Glutathione Sepharose 4 B (GE Healthcare) and Ni‐NTA Agarose (New England Biolabs) were used to purify the GST‐ and His‐tagged fusion proteins, respectively. Buffer ingredients were provided according to the manufacturer's instructions. For detection of the in vitro interaction between MUSTN1 and SMPX, 20 μL beads bound to GST‐MUSTN1 were incubated with SMPX‐His in 1 mL binding buffer (50 mM Tris–HCl, 100 mM NaCl, 10% [v/v] glycerol, 0.1% [v/v] Tween‐20, 1 mM DTT, 1 mM PMSF, 1 × cocktail protease inhibitor) at 4°C for 4 h. After washing five times with binding buffer, the beads were collected and boiled in SDS protein loading buffer for 10 min. Similarly, we examined the enrichment of MUSTN1 protein using binding beads. Finally, prey proteins were detected by immunoblotting using an anti‐GST/His antibody.

### 2.17 Binding Site Analysis and Molecular Docking

The 3D structures of the proteins were obtained from UniProt (https://www.uniprot.org/) and Alphafold database (https://alphafold.ebi.ac.uk/). MUSTN1 and SMPX were docked rigidly using the GRAMM software (http://gramm.compbio.ku.edu/). Pymol software (Version 2.4) and PDBePISA (https://www.ebi.ac.uk/pdbe/pisa/) were used for protein interaction research and visualisation analysis.

### 2.18 Statistical Analysis

All data are presented as the mean ± SD of at least three independent experiments. Statistical analyses were performed using SPSS (version 19.0; SPSS Inc., Chicago, IL, USA). Two groups were compared using Student's *t*‐test or one‐way analysis of variance followed by the least significant difference test; *p* values < 0.05 were considered statistically significant.

## 3 Results

### 3.1 MUSTN1 Is Involved in Myogenesis

**FIGURE 1:**
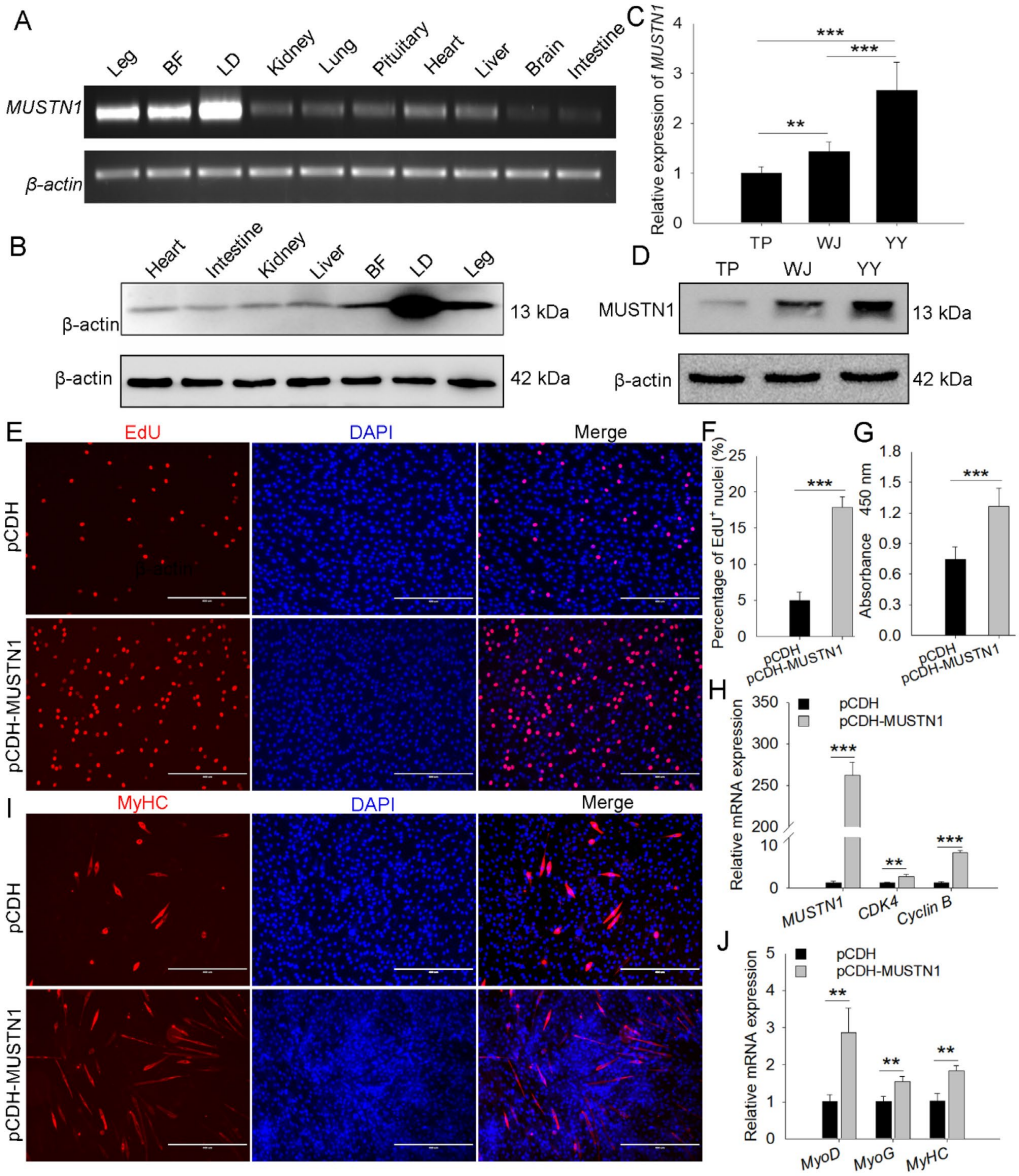
*MUSTN1* positively regulates myogenesis in pigs. mRNA (A) and protein (B) expression level analysis of *MUSTN1* in various TP tissues at the embryonic stage. *MUSTN1* mRNA (C) and protein (D) expression levels in the LD of the three pig breeds. LD, *longissimus dorsi*; BF, back fat; TP, Tibetan pig; WJ, Wujin pig; YY, Yorkshire. *n* = 6. EdU staining (E, F) and CCK8 assay (G) of proliferating cells with or without *MUSTN1* overexpression, scale bar = 400 μm. (H) mRNA expression levels of *MUSTN1* and proliferation‐related genes (*CDK4* and *Cyclin B*). (I) Representative images of MyHC immunofluorescence staining in primary pig muscle cells differentiated for 4 days. MyHC protein expression is shown in red and nuclei in blue (DAPI); scale bar = 400 μm. (J) mRNA expression levels of myogenic markers (*MyoD*, *MyoG*, and *MyHC*). Data represent the mean ± SD of at least three independent experiments. The asterisk indicates a significant difference based on the Student's *t*‐test, **p < 0.01, ***p < 0.001.

*MUSTN1* is expressed extensively in various tissues, with low expression levels in the brain and intestine and high expression in leg muscles, back fat, and LD. Furthermore, the LD showed the highest *MUSTN1* expression level, indicating that *MUSTN1* is predominantly expressed in tissues involved in muscle development and growth (Figure 1A); the protein expression results showed a similar trend (Figure 1B). The mRNA and protein expression of *MUSTN1* in LD were higher in fast‐growing YY than that in slow‐growing TP and WJ pigs (Figure 1C,D). All these results suggest that *MUSTN1* plays a role in regulating muscle development in pigs.

To investigate the function of *MUSTN1*, primary pig muscle cells were isolated and transfected. EdU staining assay showed that *MUSTN1* overexpression resulted in increased EdU positivity compared with that of the control (Figure 1E,F). CCK8 analysis showed that when *MUSTN1* was overexpressed, the absorbance value significantly increased (Figure 1G). Additionally, the expression of key proliferation marker genes *CDK4* and *Cyclin B* were significantly upregulated after *MUSTN1* overexpression (Figure 1H). We also transfected the *MUSTN1* interference fragments (siRNA‐403 with the highest interference efficiency) and pCDH‐*MUSTN1*‐pig plasmid into C2C12 cells (Figure S1A,B). The results of microscopic examination (Figure S1C,D), EdU (Figure S1E,F), CCK8 (Figure S1G,H), and proliferation markers expression analysis (Figure S1I,J) in C2C2 mouse myoblasts were similar to those in porcine muscle cells. These results suggested that *MUSTN1* accelerates myoblast proliferation. *MUSTN1* knockdown also significantly inhibited myoblast migration, and *MUSTN1* overexpression promoted migration (Figure S2A–C).

Furthermore, we assessed myoblast differentiation into myotubes by using immunofluorescence staining. After differentiation for 4 days, porcine muscle cells with overexpressed *MUSTN1* resulted in increased and long multinucleated MyHC‐positive myotubes (Figure 1I) and upregulated the expression of the myogenic marker genes (*MyHC*, *MyoG*, and *MyoD*) (Figure 1J). We also observed consistent results of marker gene expression (Figure S2D,E) and muscle tube morphology (Figure S2F–I) in C2C12 cells. These results suggested that *MUSTN1* positively regulates myoblast differentiation and myoblast fusion into myotubes in porcine and mouse myoblast.

### 3.2 MUSTN1‐KO Mice Display Decreased Muscle Fibre Cross‐Sectional Area, Muscle Mass, Exercise Endurance, and Muscle Regeneration Ability

**FIGURE 2:**
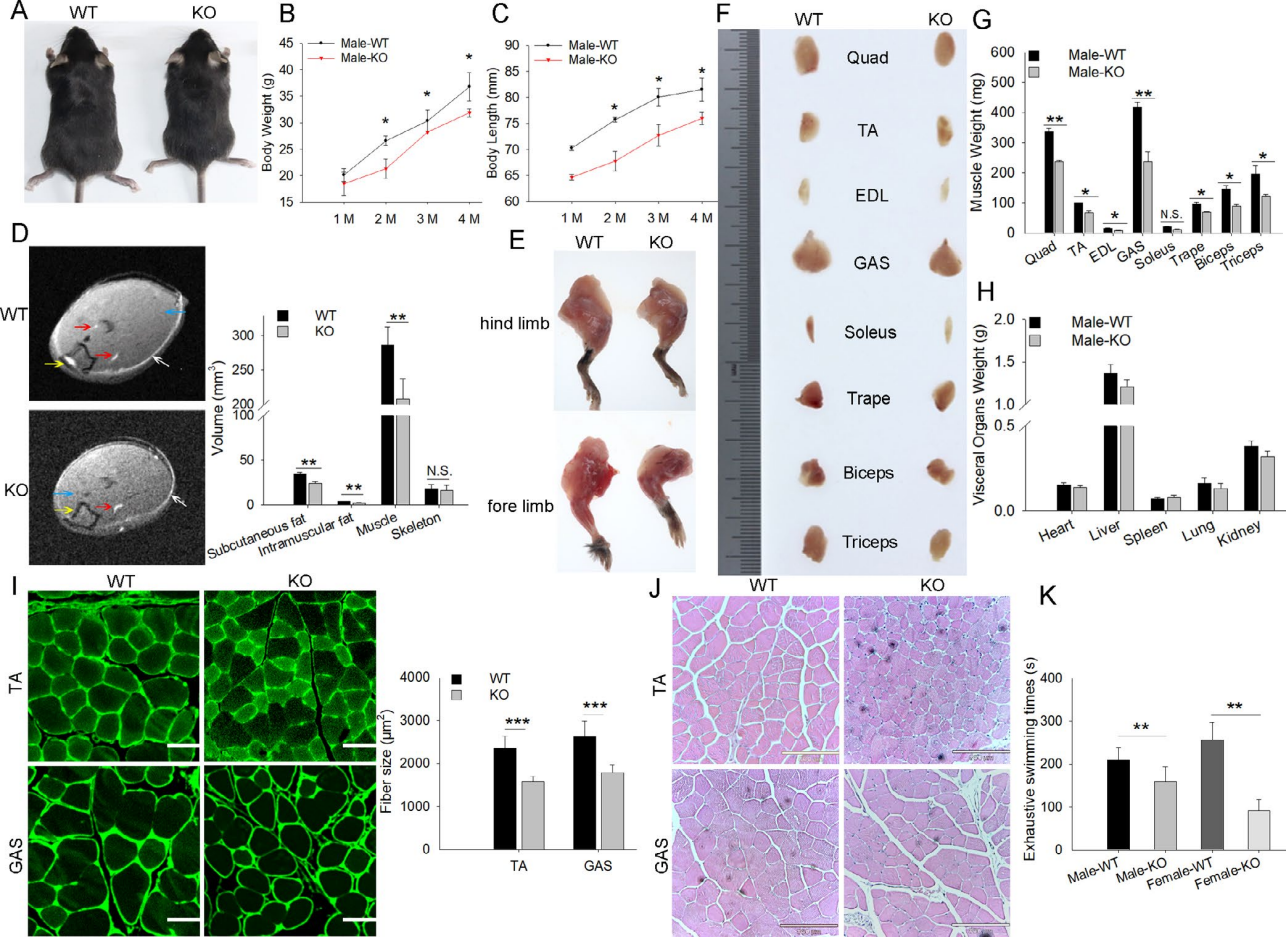
Loss of *MUSTN1* causes reduced muscle mass, cross‐sectional area, and endurance in mice. (A) Representative image of whole‐body comparison of 2‐month‐old WT and *MUSTN1*‐KO male mice. (B, C) Body weight and length measured in WT and *MUSTN1*‐KO male mice (*n* > 10); 1–4 M indicates 1–4 month‐old mice. The asterisk indicates a significant difference from 1‐month‐old. (D) Magnetic resonance imaging (MRI) of the lower hindlimbs of 2‐month‐old WT and *MUSTN1*‐KO male mice. Representative MRI images of the slices with muscle cross‐sectional area (CSA) in each limb are shown. Blue arrows indicate muscle; yellow indicates skeleton; red indicates intramuscular fat; white indicates subcutaneous fat. (E) Representative forelimb and hind limb dissection of WT and *MUSTN1*‐KO male mice. (F) Representative muscle dissections from 2‐month‐old WT and *MUSTN1*‐KO male mice. (G) Dissected muscle weights measured in 2‐month‐old WT and *MUSTN1*‐KO male mice; different letters indicate intragroup differences. (H) Dissected organ weights of WT and *MUSTN1*‐KO male mice. No significant differences between the groups. (I) Representative images of immunofluorescence staining of laminin for the tibialis anterior (TA) and gastrocnemius (GAS) muscles from 2‐month‐old WT and *MUSTN1*‐KO male mice. Laminin protein expression is shown in green; white scale bar = 100 μm. Average muscle fibre CSA (fibre size) is shown on the right. (J) Haematoxylin and Eosin (H&E) staining of TA and GAS cross‐sections of 2‐month‐old WT and *MUSTN1*‐KO male mice, scale bar = 860 μm. (K) Exhaustive swimming times of 2‐month‐old WT and *MUSTN1*‐KO male and female mice (*n* = 12 per group). Data represent the mean ± SD of at least six independent experiments. N.S., not significant. The asterisk indicates a significant difference based on the Student's *t*‐test, *p < 0.05, **p < 0.01, ***p < 0.001.

To determine the role of *MUSTN1* in muscle development in vivo, we generated *MUSTN1*‐KO mice (Figure S3A) and identified the different genotypes by using PCR (Figure S3B). The results showed that expression of *MUSTN1* was not detected in the skeletal muscles of *MUSTN1*‐KO mice (Figure S3C). *MUSTN1* was extensively expressed in various tissues of WT mice and was highly expressed in muscle and fat, similar to its expression pattern in pigs (Figure S3D). *MUSTN1*‐KO mice were smaller in size (Figure 2A) and had lower body weight and length than WT controls, and these differences were the most significant when mice were 2 months old (Figure 2B,C). MRI of the distal hind limbs revealed significantly reduced muscle mass in *MUSTN1*‐KO mice than in WT controls. Additionally, the skeletal mass of *MUSTN1*‐KO mice remained unaffected; however, it exhibited slightly decreased fat mass (Figure 2D). Further characterisation of the phenotype of *MUSTN1*‐KO mice was conducted using dissection and demonstrated that *MUSTN1*‐KO mice had stronger limbs than WT mice (Figure 2E). Next, we dissected the different muscle phenotypes (Figure 2F), and gross dissection revealed a significant reduction in skeletal muscle weight in the *MUSTN1*‐KO group compared with that of WT mice aged 1–4 months (Figure 2G and Figure S4A). Notably, visceral organs weight were not significantly different between *MUSTN1*‐KO and WT mice (Figure 2H and Figure S4B), suggesting that the decrease in body weight was primarily due to muscle growth retardation. Similarly, a delayed muscle growth phenotype was observed in the female *MUSTN1*‐KO mice (Figure S4C–F). Histological analysis showed that myofibers in *MUSTN1*‐KO mice were significantly smaller than those in WT mice, characterised by a lower cross‐sectional area in the former than in the latter (Figure 2I,J and Figure S4G), which supports the phenotypic data and indicates that the absence of *MUSTN1* leads to muscle growth and developmental retardation in mice. Exhaustive swimming exercises were performed to assess endurance; the experiment revealed a shorter exhaustive swimming time in *MUSTN1*‐KO mice than in WT control mice (Figure 2K). Notably, a similar trend was observed in male and female mice (Figure 2K). These results suggested that *MUSTN1*‐KO mice showed decreased exercise endurance.

**FIGURE 3:**
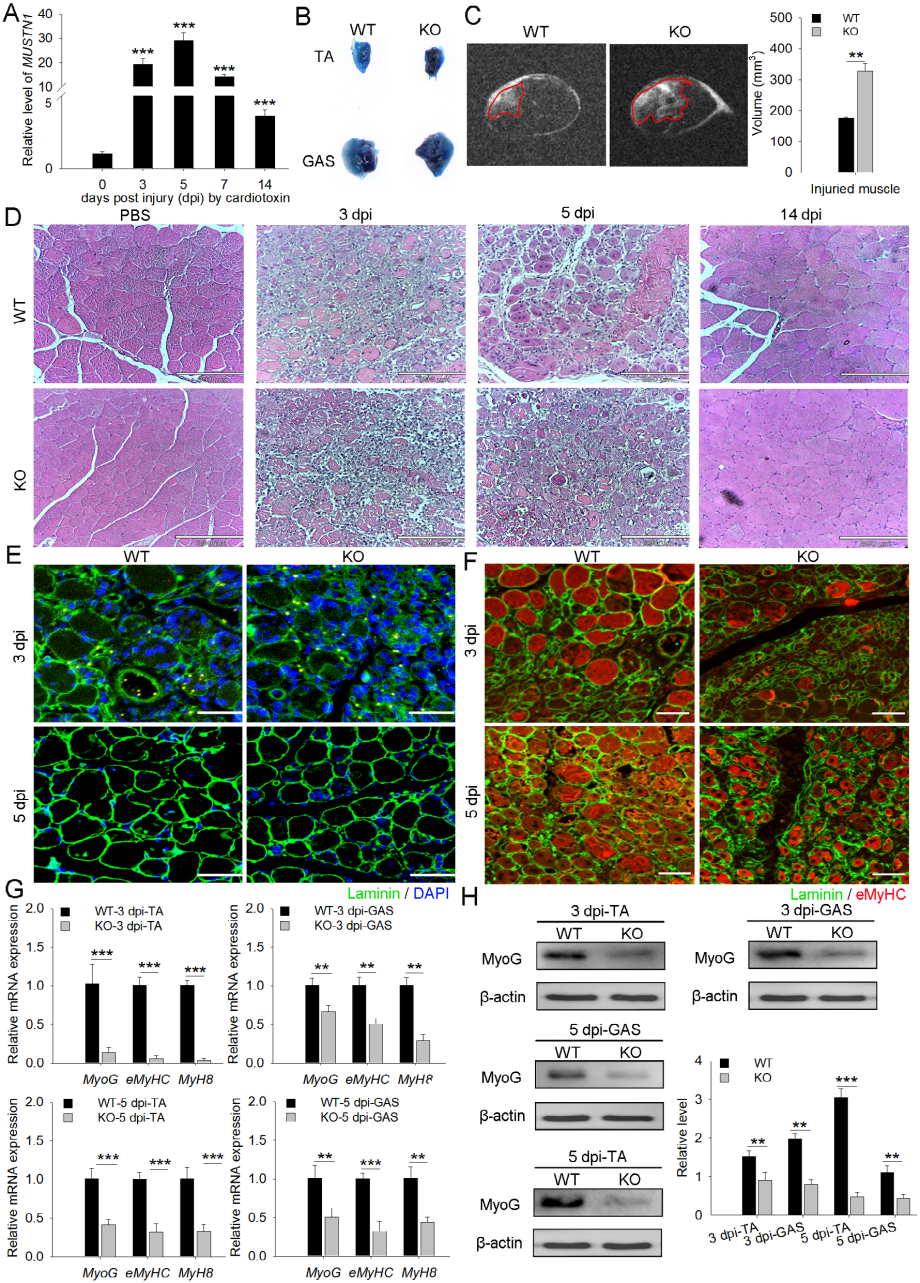
*MUSTN1* deficiency delays muscle regeneration. (A) *MUSTN1* mRNA expression in TA muscle at day 3, 5, 7, and 14 after cardiotoxin (CTX) injection. (B) Detection of myofiber damage in individual injured muscles following injection with 1% Evans blue 24 h before sampling. Comparison of TA and GAS muscles between WT and *MUSTN1*‐KO mice. Blue represents damaged muscle fibres stained using Evans blue. (C) MRI of the lower hindlimb in 2‐month‐old WT and *MUSTN1*‐KO male mice at 5 days post‐injury (dpi). Red area indicates injured muscle sites. (D) Representative images of H&E staining of the TA muscle in 2‐month‐old WT and *MUSTN1*‐KO mice at 3, 5, and 14 days post‐injury, scale bar = 890 μm. (E) Immunofluorescence analysis of laminin fibres in TA muscles of WT and *MUSTN1*‐KO mice at 3 and 5 days post‐injury; *n* = 3; green indicates laminin protein; nuclei were stained using DAPI; scale bar = 50 μm. (F) Immunofluorescence analysis of eMyHC^+^ fibres in the TA muscles of WT and *MUSTN1*‐KO mice at 3 and 5 days post‐injury; *n* = 3; eMyHC^+^ fibres represent regenerating myofibers, scale bar = 100 μm. (G) qRT‐PCR analysis of the expression of *MyoG*, *eMyHC*, and *MyH8* in TA and GAS muscles of WT and *MUSTN1*‐KO mice at 3 and 5 days post‐injury. (H) Western blot analysis of MyoG protein levels in WT and *MUSTN1*‐KO mice at 3 and 5 days post‐injury. Data represent the mean ± SD of at least three independent experiments. The asterisk indicates a significant difference based on the Student's *t*‐test, **p < 0.01, ***p < 0.001.

For our investigation of how *MUSTN1* deficiency affects muscle regeneration, the TA and gastrocnemius (GAS) muscles of *MUSTN1*‐KO and WT mice were intramuscularly injected with CTX, and the regenerated muscles were examined at different time points. The results showed that *MUSTN1* expression was upregulated in the regenerating muscles and peaked at day 5 after TA muscle injury, indicating a potential role of *MUSTN1* in muscle regeneration (Figure 3A). Myofiber membrane permeability, determined using Evans blue dye uptake, was observed in the damaged muscles of WT and *MUSTN1*‐KO mice. Myofibers in *MUSTN1*‐KO mice showed significant Evans blue dye staining, suggesting that cell membrane permeability was critically disrupted in *MUSTN1*‐KO mice (Figure 3B). MRI revealed that *MUSTN1*‐KO mice had a larger muscle injury area than WT mice (Figure 3C). H&E staining of muscle sections at different time points after injury showed that at 3 days post‐injury, control mice displayed newly formed muscle fibres with morphological integrity; however, *MUSTN1*‐KO mice did not show significant muscle fibre regeneration (Figure 3D). At 5 days post‐injury, WT mice showed most muscle fibres with centrally located myonuclei, suggesting muscle injury and regeneration. By contrast, *MUSTN1*‐KO mice showed the presence of several inflammatory cells and damaged and necrotic myofibers free of stray nuclei (Figure 3D). At day 14 after CTX injection, WT and *MUSTN1*‐KO mice exhibited tightly packed, well‐formed muscle fibres, and the muscle architecture was significantly restored, indicating that the absence of *MUSTN1* delayed but did not completely prevent regeneration (Figure 3D). Notably, the regenerated myofibers demonstrated rougher edges and smaller cross‐sectional area in *MUSTN1*‐KO mice than in WT mice, similar to those in the uninjured state (Figure 3E). To further test these data, we performed immunofluorescence staining for embryonic MyHC (eMyHC), a marker of muscle regeneration, at 3 and 5 days after CTX injection. The area of regenerated muscle fibres in *MUSTN1*‐KO mice was smaller than that in control mice at 3 days post‐injury. At 5 days post‐injury, smaller regenerated myofibers with a central nucleus were observed in *MUSTN1*‐KO muscles than in WT muscles (Figure 3F). Consistently, the mRNA levels of *MyoG*, *eMyHC*, and *MyH8* were significantly reduced in *MUSTN1*‐KO mice at 3 and 5 days after CTX injection (Figure 3G). Notably, the mRNA and protein levels of MyoG were reduced in injured muscles with MUSTN1 absence (Figure 3G,H). Collectively, these data indicated that the absence of *MUSTN1* delays skeletal muscle regeneration in adult mice, which may be related to the reduced ability of myogenic differentiation.

### 3.3 MUSTN1‐KO Inhibits Proliferation, Migration, and Differentiation of Cultured Muscle Precursor Cells

**FIGURE 4:**
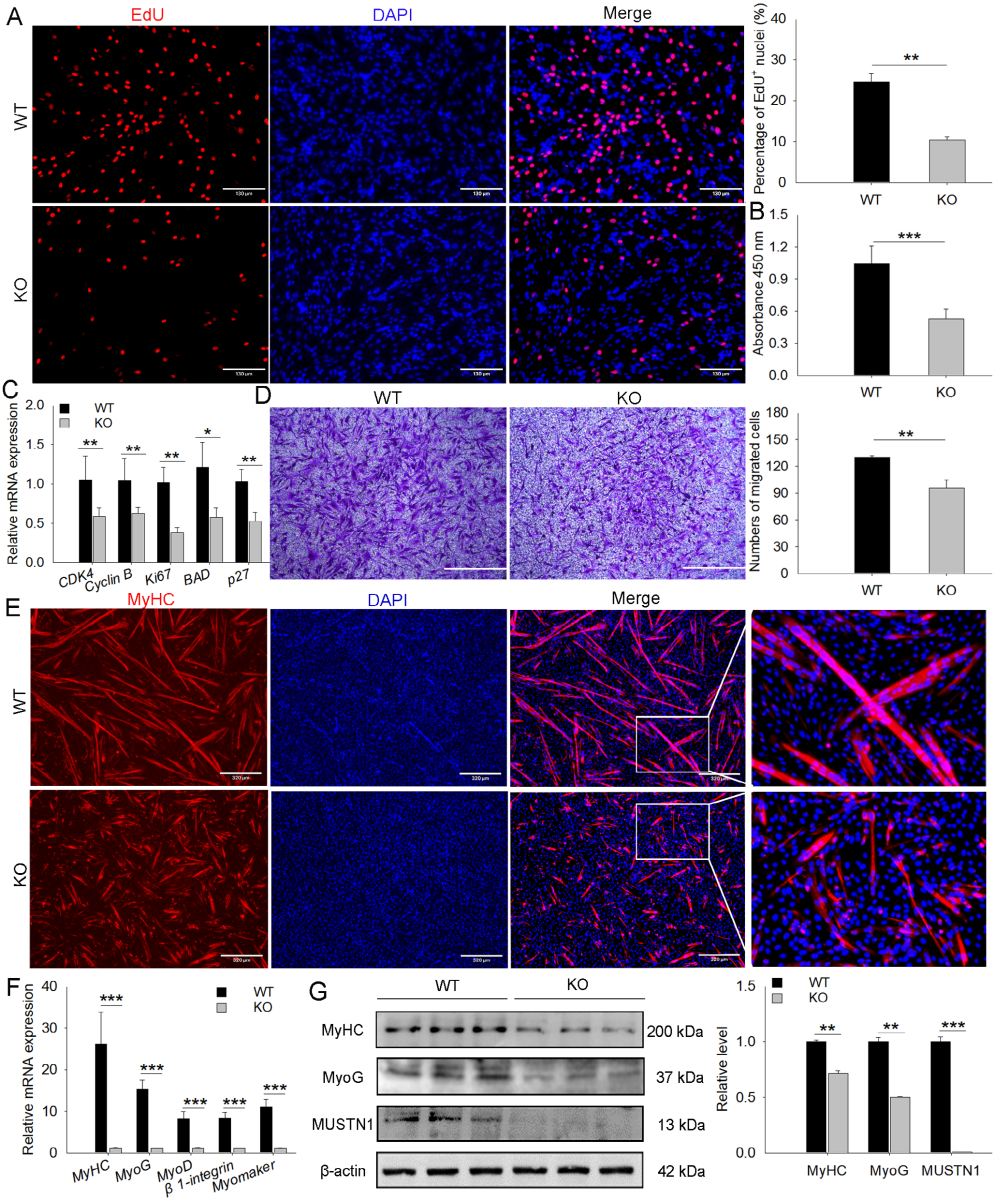
*MUSTN1*‐KO leads to impaired cell proliferation, migration, differentiation, and fusion. (A) Representative images of EdU staining and statistical analysis Scale bar = 130 μm. (B) CCK8 assay for proliferation of WT and *MUSTN1*‐KO cells. (C) qRT‐PCR analysis of the expression of proliferation (*CDK4*, *Cyclin B* and *Ki67*) and apoptosis (*BAD* and *p27*) markers. (D) Representative images of the Transwell migration assay and statistical analysis. Migrated cells were stained purple with crystal violet; bar graphs show the number of migrated cells; scale bar = 200 μm. (E) Differentiation analysis using immunofluorescence staining. MyHC protein expression is shown in red and nuclei in blue (DAPI); scale bar = 320 μm. qRT‐PCR (F) and western blot (G) analyses of myogenic marker expression. Data represent the mean ± SD of three independent experiments. The asterisk indicates a significant difference based on the Student's *t*‐test, *p < 0.05, **p < 0.01, ***p < 0.001.

We isolated muscle precursor cells from WT and *MUSTN1*‐KO mice and assessed their abilities to proliferate, migrate, and differentiate. Consistent with the results of C2C12 cells, EdU^+^ cells (Figure 4A) and absorbance values (Figure 4B) were significantly lower in *MUSTN1*‐KO cells than in WT cells. The absence of *MUSTN1* significantly downregulated the expression of proliferation marker genes (*CDK4*, *Cyclin B*, and *Ki67*) but upregulated the expression of apoptosis markers (*BAD* and *p27*) (Figure 4C). These results suggested that cell proliferation is impaired in *MUSTN1*‐KO cells. The Transwell assay showed that the loss of *MUSTN1* inhibited cell migration (Figure 4D). Most WT cells became elongated and fused and expressed the differentiation marker MyHC after differentiation induction for 4 days. By contrast, elongation, fusion, and MyHC expression were delayed in the *MUSTN1*‐KO cells (Figure 4E). The expression of differentiation and fusion marker genes was downregulated in *MUSTN1*‐KO cells (Figure 4F); protein levels showed a similar trend (Figure 4G). These results indicated that the absence of *MUSTN1* causes a delay in differentiation, which is consistent with the finding that the loss of *MUSTN1* delayed rather than completely inhibited regeneration after CTX‐induced injury (Figure 3D–F).

### 3.4 MUSTN1 and SMPX Synergistically Promote Myoblast Proliferation and Dependently Facilitate Differentiation

**FIGURE 5:**
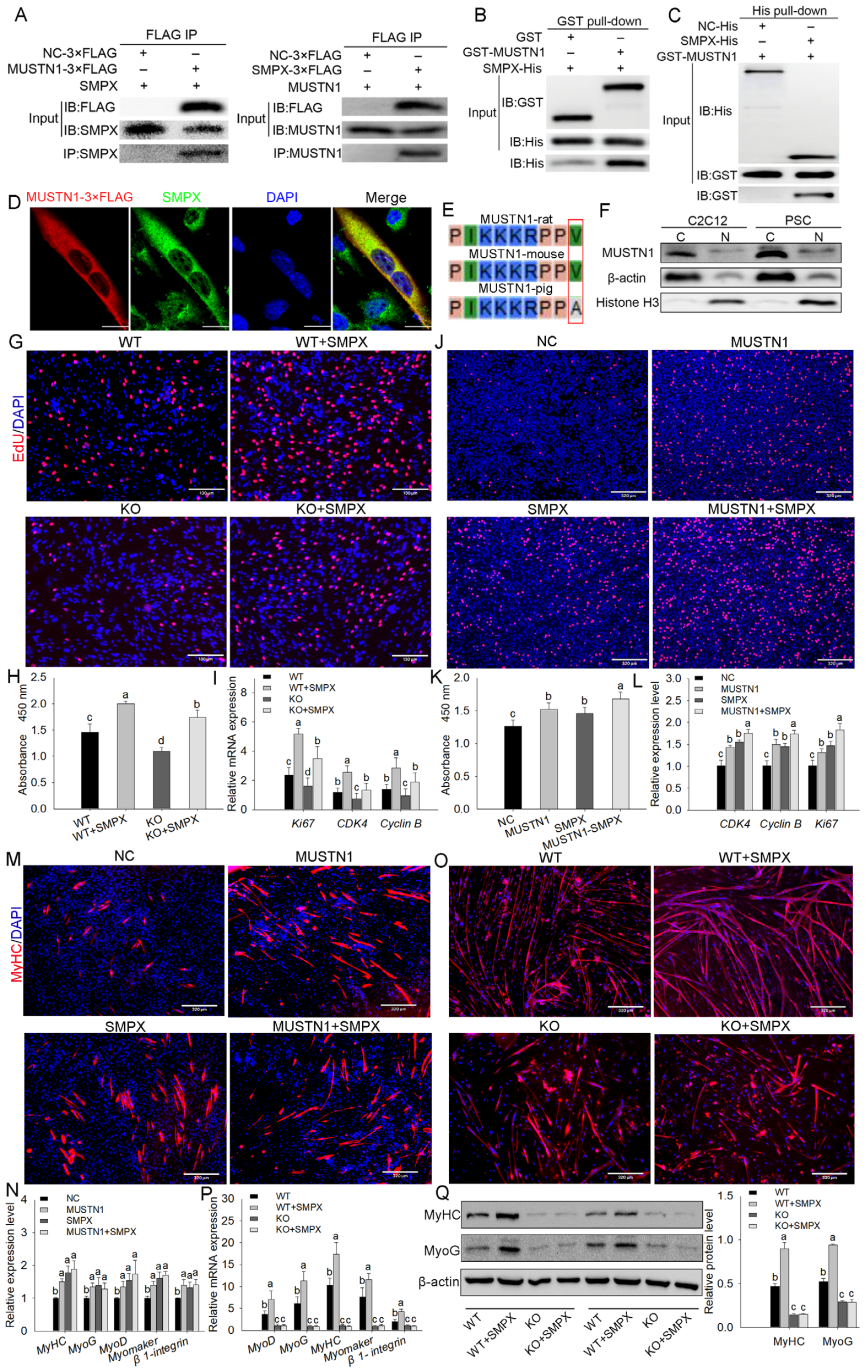
MUSTN1 and SMPX interact to facilitate myoblast proliferation and differentiation. (A) Reciprocal co‐immunoprecipitation analysis between FLAG‐tagged mouse MUSTN1 and FLAG‐tagged mouse SMPX in C2C12 cells. IB, immunoblotting; IP, immunoprecipitation. Analysis of the direct interaction between MUSTN1 and SMPX using the GST pull‐down assay (B) and His pull‐down assay (C). (D) Immunofluorescence staining for SMPX and FLAG‐tag in C2C12 cells transfected with *MUSTN1*‐3×FLAG plasmid, scale bar = 20 μm. (E) Comparison of nuclear localization sequences of MUSTN1 in different species. Red boxes indicate amino acid sites mutated in the nuclear localization sequence of pigs compared with those of rats and mice. (F) Western blotting analysis of MUSTN1 protein expression in C2C12 and porcine satellite cells (PSCs). β‐Actin and Histone H3 represent internal reference proteins for the nucleus and cytoplasm, respectively. Representative images of EdU staining (G) and CCK8 statistical analysis (H). Myoblasts were isolated from WT and KO mice and then transfected with or without pcDNA3.1‐*SMPX*‐mouse overexpression plasmid (scale bar = 130 μm). (I) qRT‐PCR analysis of proliferation markers expression Representative images of EdU staining (J) and CCK8 statistical analysis (K). pCDH‐*MUSTN1*‐mouse and pcDNA3.1‐*SMPX*‐mouse were transfected and co‐transfected into C2C12 cells; NC, negative control; scale bar = 320 μm. (L) qRT‐PCR analysis of proliferation markers expression. (M) Differentiation analysis using immunofluorescence staining. C2C12 cells were treated with separate‐ and co‐transfection of *MUSTN1* and *SMPX*. MyHC protein expression is shown in red and nuclei in blue (DAPI); scale bar = 320 μm. (N) qRT‐PCR analysis of myogenic marker expression. (O) Differentiation analysis using immunofluorescence staining. Myoblasts isolated from WT and KO mice were transfected with or without the *SMPX*‐mouse‐overexpressed plasmid (scale bar = 320 μm). qRT‐PCR (P) and western blot (Q) analyses of myogenic markers expression. Data represent the mean ± SD of three independent experiments. Different letters represent significant differences based on Student's *t*‐test.

To further investigate the molecular mechanism by which *MUSTN1* promotes muscle development, we used the Search Tool for the Retrieval of Interacting Genes online tool to predict proteins bound to MUSTN1 in mouse myoblasts. The results revealed that SMPX is a potential binding protein of MUSTN1. To confirm the MUSTN1‐SMPX association, we immunoprecipitated SMPX in C2C12 myoblasts transfected with *MUSTN1*‐3×FLAG and found that MUSTN1 and SMPX were present in the precipitate (Figure 5A). Similarly, MUSTN1 and SMPX co‐precipitated in myoblasts transfected with *SMPX*‐3×FLAG (Figure 5A). Next, we validated the direct interaction between MUSTN1 and SMPX by using a GST pull‐down assay. However, a slight non‐specific binding band was observed in the control group (Figure 5B). Subsequently, we used a heat shock protein as a negative control and found that MUSTN1 and SMPX bound directly in the His pull‐down assay (Figure 5C), indicating a direct interaction between MUSTN1 and SMPX. Immunofluorescence analysis showed that MUSTN1‐3×FLAG and endogenous SMPX co‐localised in the cytoplasm (Figure 5D). Rat‐MUSTN1‐GFP fusion protein exhibits a classical nuclear localization sequence PIKKKRPPV, which is localised in the nucleus [8]. Although MUSTN1 in rats and mice showed a similar nuclear localization sequence, there was a V to A mutation in the amino acid sequence of MUSTN1 in pigs (Figure 5E). We separated the nuclear and cytoplasmic proteins and used Histone H3 and β‐actin as the internal reference proteins. Western blotting demonstrated that endogenous MUSTN1 proteins were localised in the cytoplasm of C2C12 cells and porcine satellite cells (PSCs) (Figure 5F).

Similar to the function of *MUSTN1*, *SMPX* promoted myoblast proliferation and differentiation (Figure S5A–E). Furthermore, *SMPX* overexpression in *MUSTN1*‐deficient myoblasts still facilitated myoblast proliferation (Figure 5G–I). Co‐overexpression of *MUSTN1* and *SMPX* further accelerated C2C12 myoblast proliferation (Figure 5J–L). These results indicated that *MUSTN1* and *SMPX* synergistically promote myoblast proliferation rather than through an interdependent relationship. Notably, the co‐overexpression of *MUSTN1* and *SMPX* did not further promote myoblast differentiation (Figure 5M,N). However, in the absence of *MUSTN1*, excess *SMPX* did not restore the lower myogenic differentiation capacity (Figure 5O–Q), suggesting that *SMPX* promotes myogenic differentiation dependent on *MUSTN1*.

### 3.5 MUSTN1 Maintains Physiological Morphology of Muscle Fibres by Affecting the Stability of SMPX

**FIGURE 6:**
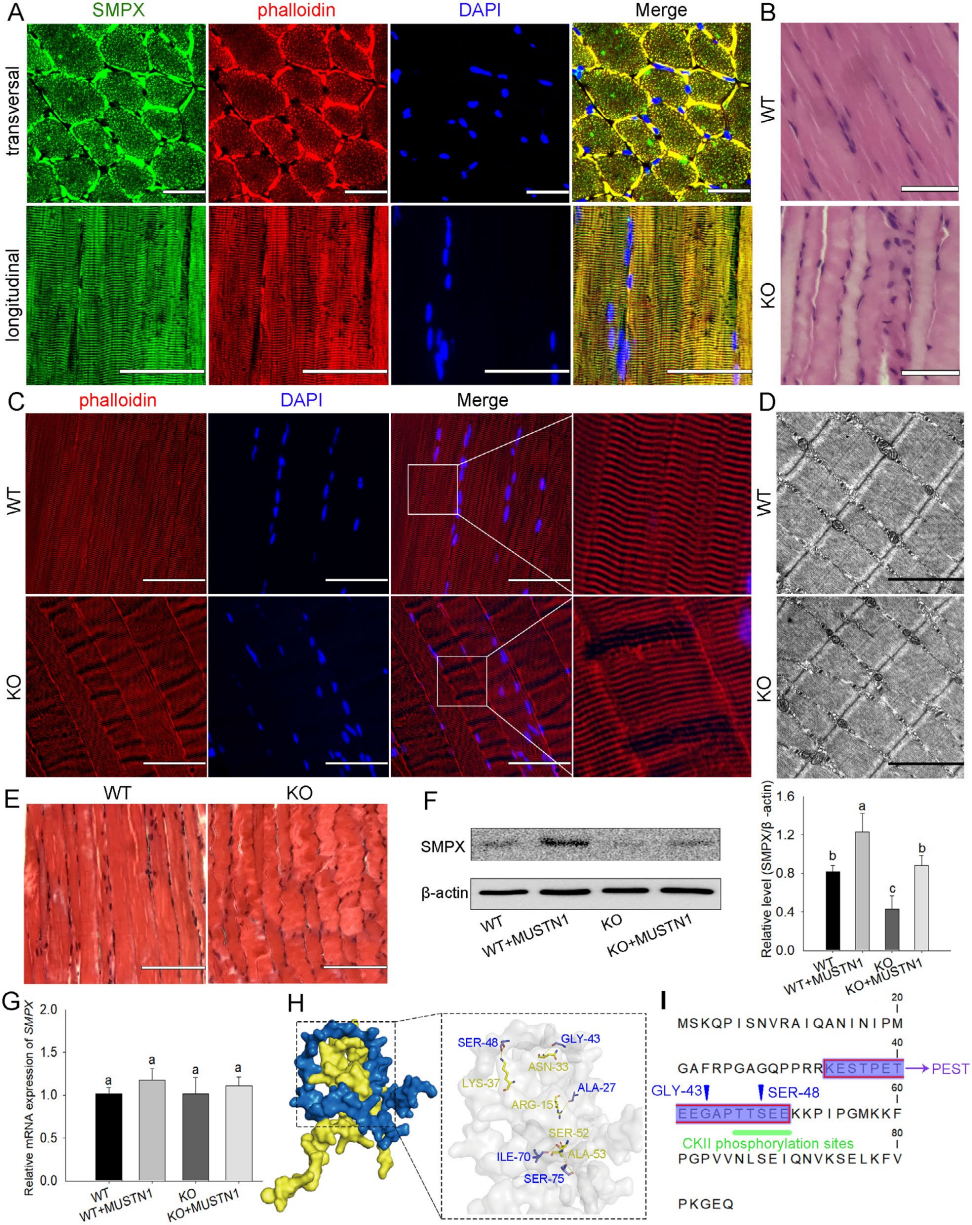
MUSTN1 Maintains morphology of muscle fibres by stabilising SMPX. (A) Immunofluorescence staining of transverse and longitudinal muscle tissues. Phalloidin‐labelled F‐Actin is shown in red, SMPX is shown in green, and nuclei are shown in blue (DAPI). Scale bar = 30 μm. (B) H&E staining of the longitudinal muscles; scale bar = 50 μm. (C) Immunofluorescence staining of longitudinal muscles. Phalloidin‐labelled F‐Actin is shown in red, and nuclei are shown in blue (DAPI); scale bar = 30 μm. (D) Transmission electron microscopy image of the muscles; scale bar = 2 μm. (E) Masson staining of longitudinal muscles, scale bar = 100 μm. (F) Western blotting and (G) qRT‐PCR analysis of SMPX expression in WT and KO myoblasts transfected with or without the *MUSTN1* overexpression plasmid. (H) Surface diagram of the docking model and interacting residues between MUSTN1 and SMPX proteins. MUSTN1, yellow; SMPX, blue. (I) Sequence diagram of SMPX protein. PEST motif, purple; CKII phosphorylation sites (TTSE and TSEE), green; interacting residues between MUSTN1 and the SMPX protein, blue arrows. Data represent the mean ± SD of three independent experiments. N.S.: not significant. Different letters indicate significant differences based on Student's *t*‐test. All TA muscle samples were obtained from 2‐month‐old male WT and KO mice.

In the transverse and longitudinal sections of the muscle tissue, SMPX was located at the same position as phalloidin‐labelled F‐actin (Figure 6A), suggesting that SMPX is an integral component of sarcomere F‐actin and maintains muscle fibre morphology. Based on the interaction between MUSTN1 and SMPX, we hypothesised that *MUSTN1* deficiency hinders the function of SMPX protein, affecting the morphology of muscle fibres. H&E staining showed that the muscle fibres of WT mice were neatly arranged, and some of the muscle fibres were curved in *MUSTN1*‐KO mice (Figure 6B). F‐actin showed a parallel and regular alignment on the longitudinal section of WT mice muscle; however, some F‐actin was not visualised in the same optical plane in *MUSTN1*‐KO mice (Figure 6C). Furthermore, transmission electron microscopy did not reveal significant abnormalities in the sarcomere structure of *MUSTN1*‐KO mice (Figure 6D), and muscle fibre fibrosis was not observed (Figure 6E). These results indicated that the absence of *MUSTN1* affects muscle mass, endurance, regenerative capacity, and muscle fibre morphology but does not obviously change sarcomere structure and muscle fibre fibrosis. Furthermore, the loss of *MUSTN1* significantly reduced SMPX protein levels; however, SMPX levels returned to normal when *MUSTN1* expression was reactivated (Figure 6F). Simultaneously, the presence or absence of *MUSTN1* did not affect *SMPX* mRNA levels (Figure 6G), which was similar to our previous transcriptome sequencing results (not shown). These results suggested that the degraded protein level of SMPX in the *MUSTN1*‐KO group is not associated with decreased transcription. The SMPX protein contains a PEST sequence and overlapping casein kinase 2 (CKII) phosphorylation sites, indicating instability [28, 29]. Molecular docking showed that the physical interactions between MUSTN1 and SMPX primarily relied on the hydrogen bonds between the five pairs of amino acids (Figure 6H). The 43rd amino acid glycine of SMPX (GLY‐43) was located at the PEST motif, and SER‐48 was located at the CKII phosphorylation binding site and the PEST motif (Figure 6I). The results indicated that the binding of MUSTN1 with SMPX at the PEST and CKII sites possibly increase stability of SMPX; thus, *MUSTN1*‐KO might cause morphological abnormalities of muscle fibres.

## 4 Discussion

Pigs are important breeding objects in agriculture, and the development and health of their skeletal muscles are directly related to the yield and quality of pork, which is one of the key factors in evaluating the growth performance and economic benefits of pigs. *MUSTN1* is a novel skeletal and muscular‐related protein that shows different expression patterns in various species and tissues; however, its expression, function, and mechanism in pigs remain poorly understood. Some studies have suggested that *MUSTN1* is specifically expressed in myocardial and skeletal muscles [30, 31]. Several studies have revealed the extensive distribution of *MUSTN1* in various tissues; notably, its expression level was high in the skeletal muscle of embryo and adult [18]. In this study, *MUSTN1* was extensively distributed in various pig tissues, especially in LD tissues, and its expression level was significantly higher in the muscle tissues of fast‐growing pigs than in those of slow‐growing pigs. These results suggest that the different expression patterns of *MUSTN1* in growing muscle tissues are related to the muscle growth rate and tissue specificity. Furthermore, *MUSTN1* accelerated pig myoblast proliferation and differentiation, indicating its positive regulatory role in pig muscle growth and development.

Myogenesis is a precisely regulated developmental process that involves complex cellular mechanisms. Myoblast precursor cells derived from somatic cells produce myoblasts, which further proliferate, migrate to target sites, fuse into multinucleated muscle tubes, and differentiate into mature muscle fibres [32]. However, the effects of *MUSTN1* on myoblast growth are controversial. *MUSTN1* knockdown promotes apoptosis of chicken skeletal muscle satellite cells [20]; Liu et al. [19] reported that *MUSTN1* did not affect cell proliferation. We demonstrated that *MUSTN1* promotes the proliferation and migration of myocytes and increases the expression of cell proliferation markers. The difference in the proliferation results compared with those of existing studies may be due to the different cell types, growth states, and *MUSTN1* expression levels. Myoblastic differentiation is essential for skeletal muscle growth, hypertrophy, and regeneration. As specific transcription factors of skeletal muscle, *MyoD* and *MyoG* are crucial in myogenic differentiation, and their reduced expression can disrupt differentiation and subsequent fusion [33]. Our study revealed that the expression of *MUSTN1* increased with myogenic differentiation and that *MUSTN1* overexpression enhanced myoblast differentiation and fusion, characterised by increased polynuclear myotubes and upregulated expression of myogenic and fusion markers. These findings suggested that *MUSTN1* promotes myoblast differentiation and fusion. These functions of *MUSTN1* during myogenesis are similar in mice and pigs.

*MUSTN1*‐KO mice were established to elucidate the function of *MUSTN1* in vivo. Notably, pigs and mice showed a similar *MUSTN1* expression pattern, with high expression in the muscle and adipose tissue. Kim et al. [34] showed that *MUSTN1* ablation results in increased glucose tolerance in male mice. However, whether *MUSTN1* plays a role in fat deposition requires further investigation. Although *MUSTN1*‐KO mice survived normally, they showed reduced weight and fibre cross‐sectional areas, and the weight loss was primarily due to decreased muscle mass rather than visceral organs. Studies have shown that *MUSTN1* affects skeletal development and regeneration [8]; however, we revealed that the bone content of the hind limbs in the WT and KO groups was not significantly different. The span of skeletal development is relatively long; therefore, the different months selected in these studies may have resulted in inconsistent results. Increased expression of *MUSTN1* in injured muscles suggested a possible link between the gene and muscle damage [35]. We confirmed that *MUSTN1*‐KO mice showed prolonged muscle regeneration, evidenced by the late formation of centralised nuclei and decreased mRNA and protein expression of muscle regeneration markers. *MUSTN1*‐KO mice showed impaired primary myoblast proliferation and differentiation, which may account for reduced muscle mass and regeneration. Muscle fibres are mainly divided into low‐twitch and fatigue‐resistant Type I and fast‐twitch Type II with easy fatiguability [36]. Kim et al. [37] found a persistent increase in Type IIb fibres and a decrease in Type I fibres in *MUSTN1*‐KO mice at both 2 and 4 months, consistent with our observation of reduced exercise endurance in KO mice. Female mice performed better than male mice in exercise endurance. In further research, we plan to focus on sex‐induced differences in muscle development.

Lombardo et al. [8] reported that GFP‐MUSTN1 was localised in the nucleus of preosteoblastic MC3T3 cells. However, our western blotting showed that endogenous MUSTN1 was localised in the cytoplasm of mouse and pig myoblasts after nucleoplasmic separation. Furthermore, we identified SMPX as a small protein that interacts with MUSTN1. SMPX is localised in the cytoplasm [38], and the colocalization results of SMPX and MUSTN1 further confirmed the presence of MUSTN1 in the cytoplasm. Our findings were inconsistent with those of Lombardo et al. [8]; which may be due to different cell systems and states or because a large GFP tag affected the localization of the small MUSTN1 protein.

*SMPX* is highly expressed in the heart and skeletal muscles and is key in maintaining the costameric cytoskeleton and cell shape [39]. We demonstrated that *SMPX* positively regulated myoblast proliferation and differentiation, endowing this protein with a novel function. Additionally, we found that *SMPX* promoted myoblast proliferation in the absence of *MUSTN1*, suggesting that *MUSTN1* and *SMPX* are relatively independent and not based on their interactions in the regulation of myoblast proliferation. Notably, *MUSTN1*‐KO inhibited the promoting effect of *SMPX* on myoblastic differentiation. These results indicated that the disintegration of the MUSTN1‐SMPX complex disrupts some of the physiological effects of SMPX that are dependent on MUSTN1. The signalling pathway of the MUSTN1‐SMPX complex that regulates skeletal muscle development requires further investigation.

SMPX plays a crucial role in protecting plasma membranes from mechanical stress [28]. We found that SMPX was a component of sarcomere. Notably, the muscle fibres in WT mice showed F‐actin‐labelled and typical transverse structures, and some F‐actin in *MUSTN1*‐KO mice were not in the same optical plane; the sarcomere structure did not show significant abnormalities. Because of the interaction between MUSTN1 and SMPX, we suggest that breakage of the MUSTN1‐SMPX complex incapacitates muscle fibres to withstand mechanical stress, resulting in myofibers bending slightly. These results are similar to the defective organisation of muscle fibres in *SMPX*‐mutant zebrafish [40]. Palmer et al. [41] reported that *SMPX* overexpression affected cytoskeletal dynamics and enhanced cell fusion in C2C12. Furthermore, we found that MUSTN1 affected SMPX protein levels but not *SMPX* transcription. The PEST sequence and overlapping CKII served as indicators of SMPX protein instability [28]. On the one hand, the interaction between MUSTN1 and SMPX occurred at the phosphorylation site of CKII, and we speculated that MUSTN1 competitively occupies the site with CKII, resulting in SMPX remaining stable without CKII phosphorylation. On the other hand, the interaction between MUSTN1 and SMPX also occurred at PEST, which may result in the PEST sequence not being exposed, preventing the degradation of SMPX. Therefore, we speculate the molecular mechanism by which MUSTN1 maintains SMPX stability. Since MUSTN1 and SMPX have low molecular weights, there are still some difficulties in confirming this phenomenon.

Currently, most reports on *MUSTN1* in muscles are confined to the realm of high‐throughput sequencing and gene expression data, with only a handful offering preliminary cellular validation, primarily in poultry. This study revealed that *MUSTN1* is a novel positive regulator of myogenesis in mice and pigs and that it regulates myoblast proliferation and differentiation. We also observed that, after nucleocytoplasmic separation, MUSTN1 is localised in the cytoplasm of pig myoblasts. *MUSTN1*‐KO mice displayed decreased growth rate, muscle mass, fibre cross‐sectional area, exercise endurance, and delayed muscle repair and regeneration. Mechanistically, MUSTN1 interacts with SMPX, synergistically promotes myoblast proliferation, and inter‐dependently facilitates differentiation. This is a novel mechanism by which *MUSTN1* regulates muscle development. Additionally, MUSTN1 is crucial for maintaining the stability of SMPX and the morphology of the muscle fibres. In conclusion, our paper not only reveals the important role of *MUSTN1* in muscle development at both in vitro and in vivo levels, but also identifies its localization in cells and the mechanism by which it interacts with SMPX to regulate muscle development, suggesting that *MUSTN1* shows potential for treating muscle disease and improving animal meat production.

## Author Contributions

All authors have read and agreed to the published version of the manuscript. Y.F. and X.H. conceived and designed the experiments. Y.F. and X.H. performed the experiments. Y.F., X.H., J.N., B.Z., and H.Z. analysed and interpreted the data. P.S., Y.Z., and H.Z. funded and supervised the research. X.H., Y.F., and H.Z. wrote the manuscript.

## Ethics Statement

All experimental animal procedures were performed in accordance with the China Agricultural University's regulations for animal care and handling. The China Agricultural University Laboratory Animal Welfare and Animal Experimental Ethics Committee (permit number: AW80203202‐1‐1) authorised the study and approved the protocol.

## Conflicts of Interest

The authors declare no conflicts of interest.

## Supporting information


**Data S1** Supporting Information.

## Data Availability

The data that support the findings of this study are available from the corresponding author upon reasonable request.
