# Supplementary material for: MUSTN1 Interaction With SMPX Regulates Muscle Development and Regeneration
Source: Cell Prolif. 2025 Jan 19;58(6):e13809. doi: 10.1111/cpr.13809 (PMC12179556; doi:10.1111/cpr.13809)
Supplement: Supplementary file 1 — Data S1 Supporting Information. [file CPR-58-e13809-s001.docx]

**SUPPLEMENTAL INFORMATION**


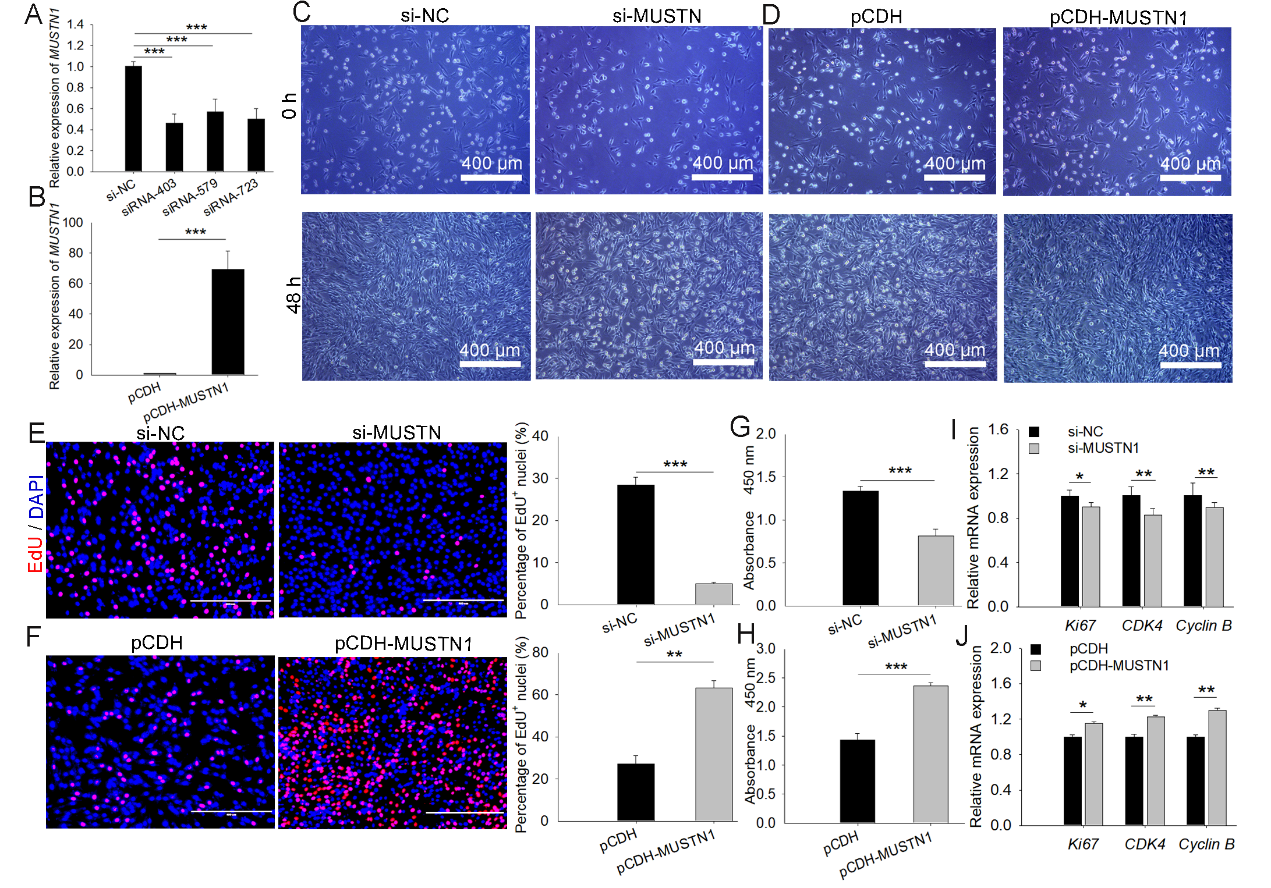


**Supplementary Fig. 1.** Microscopic images show that *MUSTN1* facilitates myoblast proliferation. (A, B) *MUSTN1* expression in C2C12 cells transfected with siRNA or an overexpression plasmid. (C, D) Microscopic images of proliferating cells. C2C12 cells were transfected for different time points (0 and 48 h); scale bar = 400 μm. (E, F) EdU staining and statistical analysis of cell proliferation. Nuclei were stained with DAPI, scale bar = 400 μm. (G, H) CCK8 assay of proliferating cells with *MUSTN1* interference or porcine-*MUSTN1* overexpression. (I, J) mRNA expression levels of proliferation-related genes. NC, negative control. The data represent the mean ± SD of three independent experiments. The asterisk indicates a significant difference based on the Student’s t-test, **p* < 0.05, ***p* < 0.01, ****p* < 0.001.


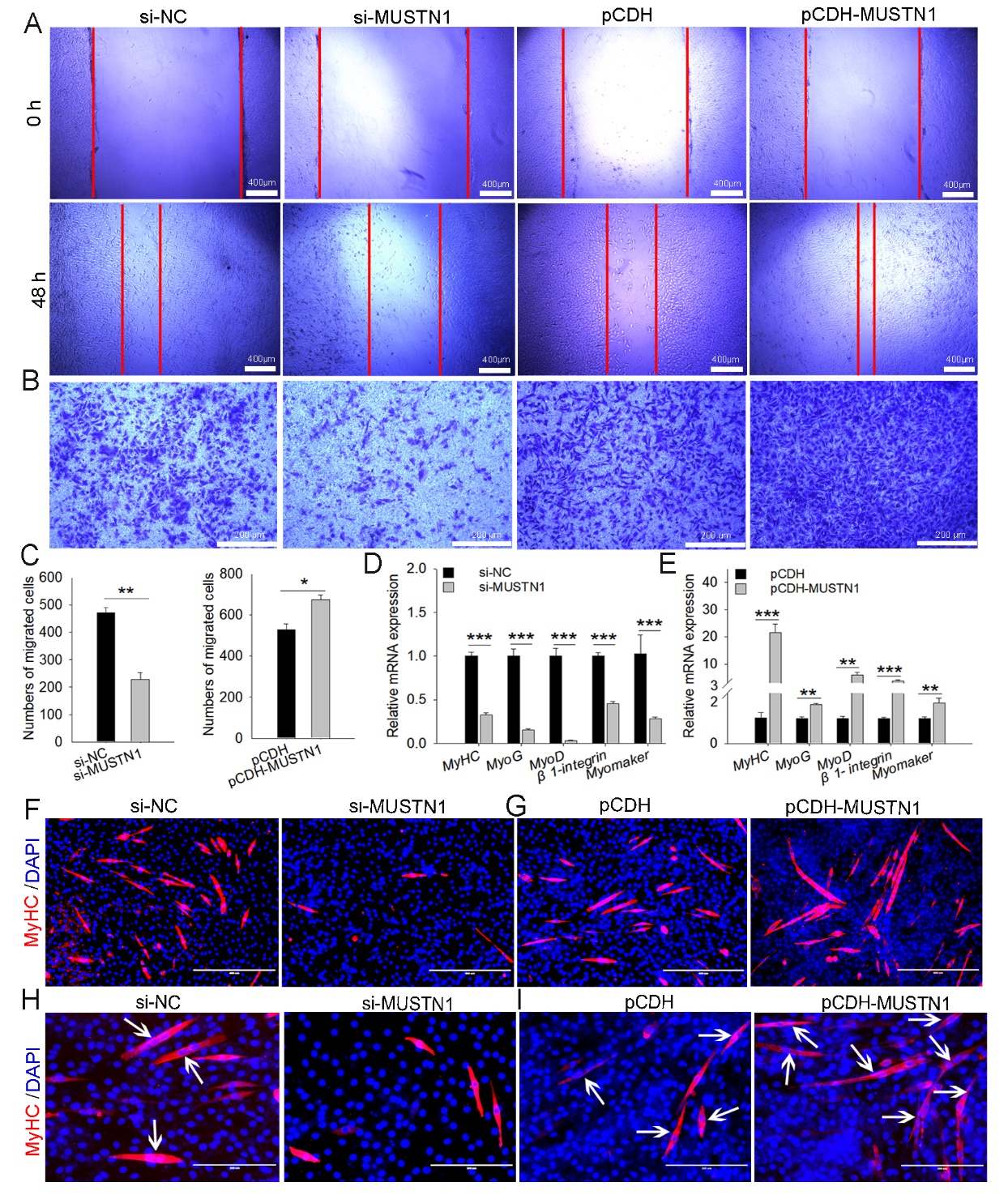


**Supplementary Fig. 2.** *MUSTN1* promotes migration, differentiation and fusion of mouse C2C12 myoblasts. (A) Representative photographs of C2C12 cell migration after wound healing at 0 h and 48 h; scale bar = 400 μm. (B) Transwell assay of migrating cells with knockdown or overexpressed *MUSTN1*. Purple indicates migrated cells stained with crystal violet, scale bar = 200 μm. (C) Bar graphs show the numbers of migrated cells. mRNA expression of myogenic (*MyHC*, *MyoG,* and *MyoD*) and fusion (*Myomaker* and *β1*-*integrin*) marker genes in C2C12 cells with *MUSTN1* knockdown (D) or overexpression (E). The cells were differentiated for 4 days. Representative photographs of MyHC immunofluorescence staining in *MUSTN1* knockdown (F) or overexpression (G) cells differentiated for 4 days. MyHC protein expression is shown in red and nuclei are shown in blue (DAPI); scale bar = 400 μm. (H, I) Myoblast fusion analysis based on immunofluorescence staining for MyHC. Arrows represent multinucleated myotubes (scale bar = 200 μm). NC, negative control. The data represent the mean ± SD of three independent experiments. The asterisk indicates a significant difference based on the Student’s *t*-test, **p* < 0.05, ***p* < 0.01, ****p* < 0.001.

**
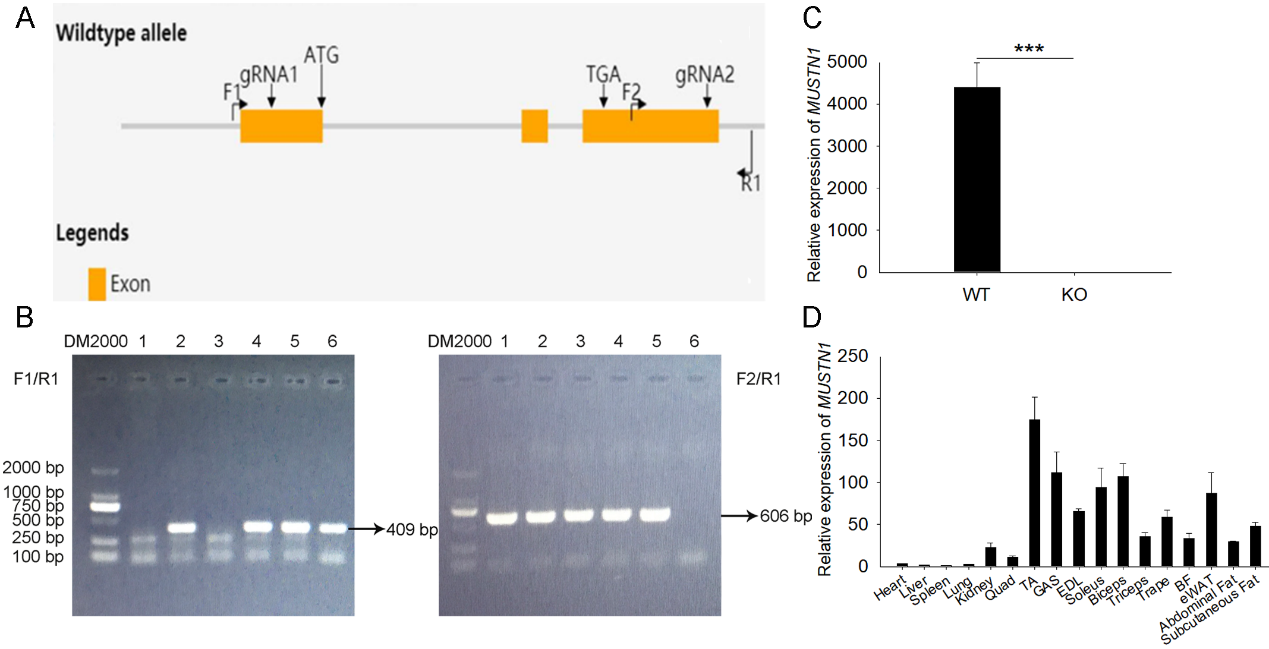
**

**Supplementary Fig. 3.** Construction of *MUSTN1* knockout mice. (A) Schematic diagram of the *MUSTN1* deletion in the mouse genome. (B) Genotypes of knockout (KO) and wild-type (WT) mice were identified using PCR. Numbers 1–6 indicate six mice; WT mice had an amplicon of 606 bp, KO mice had an amplicon of 409 bp, and heterozygous mice had both bands. (C) mRNA expression levels of *MUSTN1* in TA muscles of 2-month-old WT and KO mice; n = 6. Data represent mean ± SD. The asterisk indicates a significant difference based on Student’s *t*-test, ****p* < 0.001. (D) qRT-PCR analysis of *MUSTN1* expression in different tissues of 2-month-old WT mice, n = 6. Quad, quadriceps; TA, tibialis anterior; GAS, gastrocnemius; EDL, extensor digitorum longus; Trape, Trapezius; BF, back fat; eWAT, epididymis white fat.

**
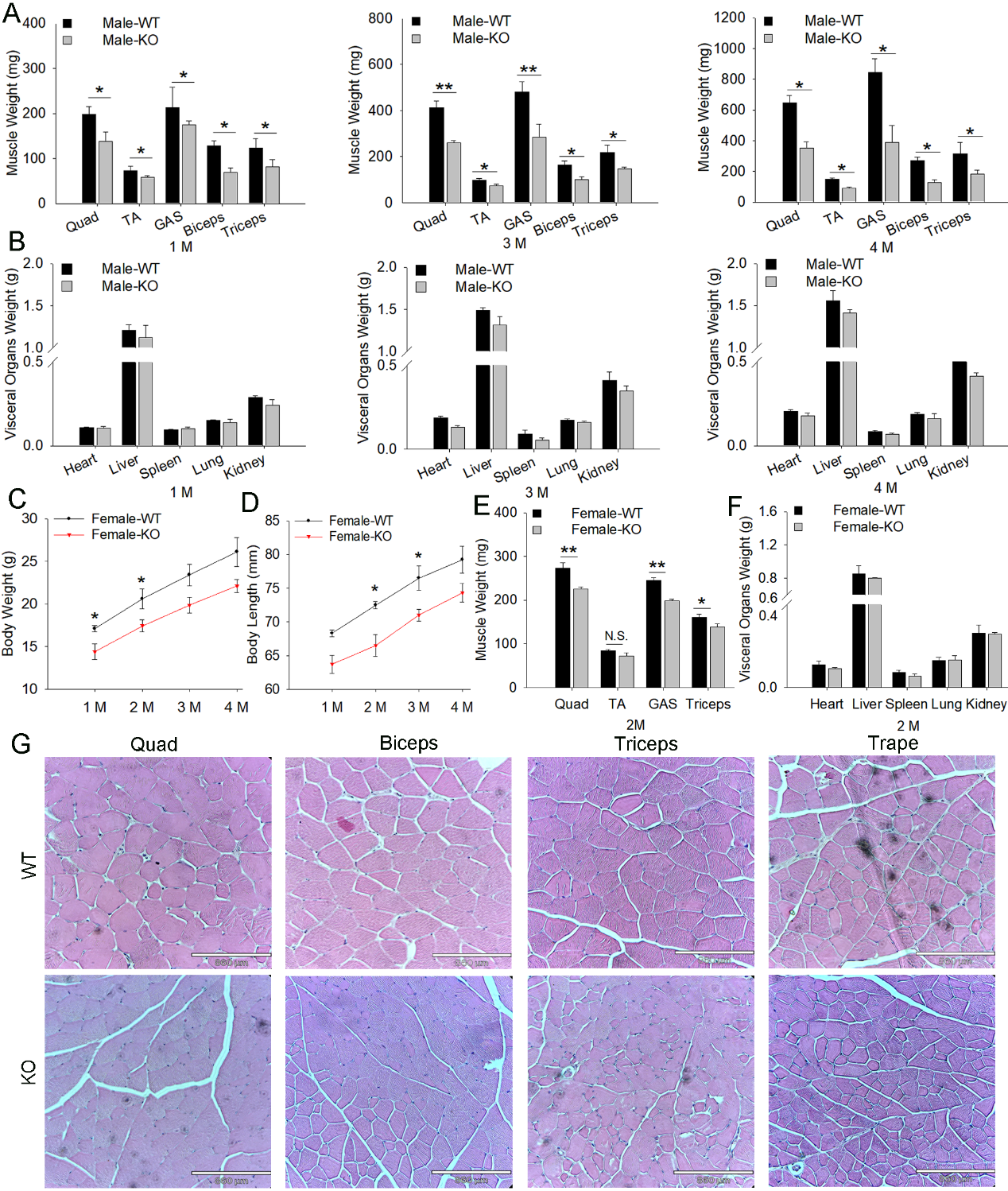
**

**Supplementary Fig. 4.** *MUSTN1*-KO mice show retardation of muscle growth. (A) Dissected muscle weights of WT and *MUSTN1*-KO male mice; 1, 3, and 4 M indicate 1, 3, and 4-month-old, respectively. (B) Dissected organ weights of WT and KO male mice. There were no significant differences between the groups. Body weight (C) and body length (D) of female WT and KO mice; 1–4 M indicates 1–4 month-old mice. The asterisk indicates a significant difference from 1-month-old. The weights of muscles (E) and visceral organs (F) of 2-month-old female WT and *MUSTN1*-KO mice. (G) H&E staining of muscle cross-sections of 2-month-old WT and KO male mice; scale bar = 860 μm. n = 6–10 mice per genotype. The data represent the mean ± SD of at least six independent experiments. N.S., not significant. The asterisk indicates a significant difference based on the Student’s *t*-test, **p* < 0.05, ***p* < 0.01.

**
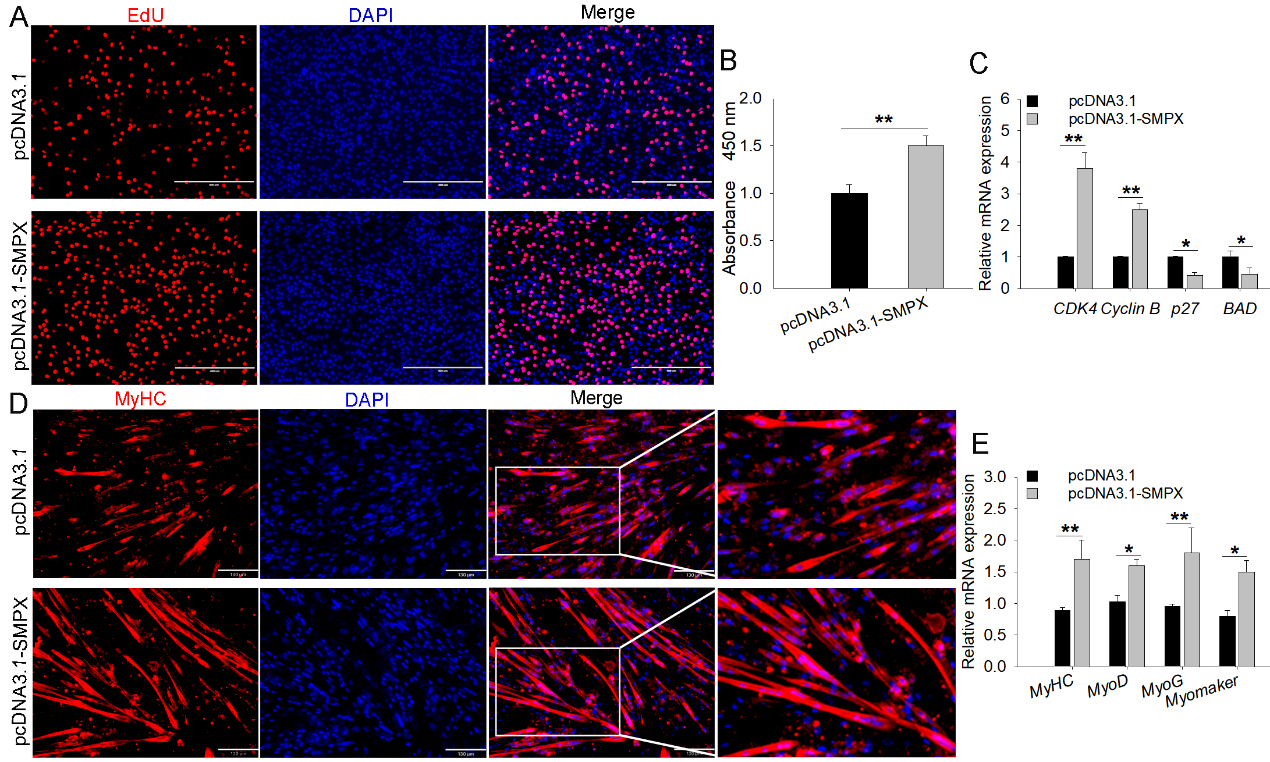
**

**Supplementary Fig. 5.** *SMPX* promotes myoblast proliferation and differentiation. (A) Representative photographs of EdU staining of proliferating cells (scale bar, 400 μm). (B) CCK8 assay for proliferation of C2C12 cells transfected with pcDNA3.1 or pcDNA3.1-*SMPX*-mouse plasmids. (C) qRT-PCR analysis of the expression of proliferation and apoptosis markers. (D) Differentiation analysis using immunofluorescence staining. The control and overexpressed cells were differentiated for 4 days. MyHC protein expression is shown in red and nuclei are shown in blue (DAPI); scale bar = 130 μm. (E) qRT-PCR analysis of myogenic markers expression. The data represent the mean ± SD of three independent experiments. The asterisk indicates a significant difference based on the Student’s *t*-test, **p* < 0.05, ***p* < 0.01.

**Supplementary Table 1.** **The primer sequences for SqRT-PCR and qPCR**

| Species | Genes | Primer sequences (5′ to 3′) |
| --- | --- | --- |
| Pig  Mouse | *MUSTN1*  *β-actin*  *GAPDH*  *MyH8*  *eMyHC*  *Ki67*  *Cyclin B* | F: CAGGAAGCCCCCATCAAGA  R: AGTCCGAGCACGGCTGAAC  F: GCCAACCGTGAGAAGATGACT  R: GTGACCCCATCCCCAGAGT  F: CACCATCTTCCAGGAGCGAG  R: CCCTTCAAGTGAGCCCCG  F: GGGCTGTACCAGAAGTCAGC  R: AAAGGGCTGACACAGTTTGG  F: TCAGGATTCGGAGGAGCAGG  R: CTTCTTGTCCAGAGCGGCAG  F: ATCATTGACCGCTCCTTTAGGT  R: GCTCGCCTTGATGGTTCCT  F: AATACCTACAGGGTCGTGAAGTGA  R: GCTGTATCATCTTCTTGGGCAC |
|  | *CDK4*  *p27* | F: GCTGCTACTGGAAATGCTGACC  R: AGCCTTGGGGGGAAACAGA  F: CAGGCAAACTCTGAGGACCG  R: TCGGGGAACCGTCTGAAAC |
|  | *BAD* | F: GCTTAGCCCTTTTCGAGGAC  R: GATCCCACCAGGACTGGAT |
|  | *MyoD* | F: CGAGCACTACAGTTGGCGACTAAGAT  R: GCTCCACTATGCTGGACAGGCAGT |
|  | *MyoG*  *MyHC*  *Myomaker* | F: CCATCCAGTACATTGAGCGCCTACA  R: ACGATGGACGTAAGGGAGTGCAGAT  F: CAAGTCATCGGTGTTTGTGG  R: TGTCGTACTTGGGCGGGTTC  F: ATCGCTACCAAGAGGCGTT  R: CACAGCACAGACAAACCAGG |
|  | *β1-integrin*  *MUSTN1*  *SMPX*  *β-actin*  *GAPDH* | F: TTACAAGAGTGCCGTGACAACTG  R: GACTAAGATGCTGCTGCTGTGAG  F: CCCCTGTGAAGGAAGAAGACC  R: CGGTTGCGGCTGAATACA  F: CAGCCTCCCAGAAGGAAAGA  R: TCTCAGACAAGTTGACAACAGGTC  F: ATCTGGCACCACACCTTCTACA  R: AAGGTCTCAAACATGATCTGGGT  F: TGCTGAGTATGTCGTGGAGTCT  R: ATGCATTGCTGACAATCTTGAG |

**Supplementary Table 2.** **The primer sequences for overexpression plasmid construction**

| Primer name | Primer sequences (5′ to 3′) |
| --- | --- |
| pCDH-*MUSTN1*-pig  pCDH-*MUSTN1*-mouse | F: ttcgaatttaaatcgCCACCATGTCCCAGGAA |
|  | R: tcgcagatccttcgcCACTTCTCAGCCAAAGACACT  F: ttcgaatttaaatcgATGTCCGAGGCTGGCACTCC  R: tcgcagatccttcgcGCCAAACACGCTCTTGGCTG |
| 3×FLAG-*MUSTN1*-mouse  3×FLAG-*SMPX*-mouse | F: tagcgtttaaacttaagcttgccaccATGTCCGAGGCTGGCACTCC |
|  | R: gctggatatctgcagaattcGCCAAACACGCTCTTGGCTG  F: tagcgtttaaacttaagcttgccaccATGTCGAAGCAGCCAATTTCC  R: gctggatatctgcagaattcCTGTTCACCTTTGGGGACAAAT |

**Supplementary Table 3. The sequences of *MUSTN1* siRNA fragments**

| siRNA (mouse) | Oligo sequences (5′ to 3′) |
| --- | --- |
| si-NC | CGAGCUCGAAUCACGGUCATT |
|  | UCAGGCUGAAGACCGGAUCTT |
| si-430 | GAGGGACCCUGGCCAAGAATT |
|  | UUCUUGGCCAGGGUCCCUCTT |
| si-579 | GCCAAGAGCGUGUUUGGCUTT |
|  | AGCCAAACACGCUCUUGGCTT |
| si-723 | GCCCUUGGAAACACCAAUATT |
|  | UAUUGGUGUUUCCAAGGGCTT |

**Supplementary Table 4.** **The gRNAs and primers for *MUSTN1^-/-^* mouse construction**

| Name | Sequences (5′ to 3′) |
| --- | --- |
| gRNA-1 | ACAGCTGCTTACTCGACGGTGGG |
| gRNA-2 | ACACGTAATGCAATGCCCGCAGG |
| KO-F1  WT-F2  R1/R2 | GCTAAACAAACTCAGAGCATCCAC |
|  | ATAAAGAGTATGCCCAATGTCCCC  AAGGAGTAAGGATAATGGGCTTGG |

**Supplementary Table 5.** **The primer sequences for pull-down assay**

| Primer name | Primer sequences (5′ to 3′) |
| --- | --- |
| pGEX-4T-1-MUSTN1  pET-30a-SMPX | F: atctggttccgcgtggatccATGTCCGAGGCTGGCACTC |
|  | R: tcacgatgcggccgctcgagGCCAAACACGCTCTTGGCT |
|  | F: cccagatctgggtaccATGTCGAAGCAGCCAATTTCC  R: tcgagtgcggccgcaagcttCTGTTCACCTTTGGGGACAAAT |
